# Uncovering the physiology and distribution of thallium in Tl-hyperaccumulating and Tl-sensitive populations of *Biscutella laevigata* L.

**DOI:** 10.1093/aob/mcae115

**Published:** 2024-09-28

**Authors:** Mirko Salinitro, Sandrine Isnard, Dennis Brueckner, Kathryn M Spiers, Mark G M Aarts, Amelia Corzo Remigio, Antony van der Ent

**Affiliations:** Department of Biological, Geological and Environmental Sciences, University of Bologna, Bologna, Italy; AMAP, Université de Montpellier, IRD, CIRAD, CNRS, INRAE, Montpellier, France; Deutsches Elektronen-Synchrotron DESY, Hamburg, Germany; Deutsches Elektronen-Synchrotron DESY, Hamburg, Germany; Laboratory of Genetics, Wageningen University and Research, Wageningen, The Netherlands; Centre for Water in the Minerals Industry, Sustainable Minerals Institute, The University of Queensland, Brisbane, Queensland, Australia; Laboratory of Genetics, Wageningen University and Research, Wageningen, The Netherlands

**Keywords:** Bioconcentration, phytoextraction, intraspecific variation, metal tolerance, synchrotron-based micro-X-ray fluorescence, *Biscutella laevigata* L

## Abstract

**Background and Aims:**

Thallium (Tl) is extremely toxic to all life forms and is an emerging pollutant. Plants in the Brassicaceae family, including edible crops, have an enhanced capacity for Tl accumulation, even from soils with low thallium concentration. The most extreme Tl hyperaccumulator is *Biscutella laevigata*, capable of attaining >32 000 μg Tl g^−1^ dry weight (DW) in its leaves.

**Methods:**

*Biscutella laevigata* from a non-metallicolous accession (Feltre, Italy) and a metallicolous accession (Les Malines, France) were subjected to a dosing experiment in hydroponics (0, 5 and 30 μm Tl), followed by synchrotron-based micro-X-ray fluorescence analysis to elucidate tissue- and cellular-level Tl distribution.

**Key Results:**

Flow cytometric data on the two accessions showed that the Feltre accession has a genome size twice of that of the Les Malines accession (256 and 125 pg per 2*C*, respectively), suggesting that they are phylogenetically distant populations. The Feltre accession did not accumulate Tl (125 μg Tl g^−1^ DW on average in leaves) at the 5 µm Tl dose level, whereas the Les Malines accession had a mean of 1750 μg Tl g^−1^ DW, with peaks of 24 130 μg Tl g^−1^ DW, at the 30 µm Tl dose level. At 30 µm Tl, the non-metallicolous accession did not grow, and at 5 µm Tl it showed reduced biomass compared with the metallicolous one. In the Les Malines accession, the synchrotron-based micro-X-ray fluorescence analysis revealed that Tl was localized in the vacuoles of epidermal cells, especially underneath trichomes and in trichome basal cells. Thallium also occurred in solid crystalline deposits (3–5 µm in size, ~40 wt% Tl) that were found mainly in foliar margins and under trichome bases.

**Conclusions:**

*Biscutella laevigata* is an attractive model for studying Tl hypertolerance and hyperaccumulation on account of the extreme expression of this trait and its marked intraspecific variability.

## INTRODUCTION

Thallium (Tl) is the most toxic element known on Earth (Lennartson, 2015), given that even small doses (8 mg kg^−1^ body weight) can be lethal to humans (Moeschlin, 1980). Despite its potential toxicity to ecosystems and humans, this element is often given little thought by the regulatory authorities (Campanella *et al.*, 2019). Thallium is a relatively rare metal, with an average concentration of 0.9 µg g^−1^ in the upper continental crust (Rudnick and Gao, 2003; Karbowska, 2016), but geochemically anomalous and polluted areas with elevated Tl are known worldwide, where this element is a major environmental contaminant (Xiao *et al.*, 2012; Liu *et al.*, 2018). The chemical behaviour of Tl resembles that of chalcophile elements on the one hand (Cu, Pb, Zn, Hg, Sb and As) and that of the alkali metals (K, Rb, Cs and Na) on the other (Liu *et al.*, 2018). Thallium occurs in two oxidation states: monovalent Tl(I) being the most common, and trivalent Tl(III), which is rare in nature and poorly bioavailable, but 50 000-fold more toxic than the former (Ralph and Twiss, 2002). In general, the mineral phases that can host notable amounts of Tl belong mainly to sulfide ore deposits, among them sphalerite with ≤1800 µg Tl g^−1^ (Henjes-Kunst *et al.*, 2017), galena (George *et al.*, 2016), pyrite and other Fe sulfides (George *et al.*, 2019) and jarosite (Garrido *et al.*, 2020). Primary sources of Tl in the environment are natural volcanogenic hydrothermal deposits or related mining activities. In particular, decommissioned mine sites can represent one of the major sources of Tl in the environment (Barago *et al.*, 2023).

In contaminated areas (both natural and anthropogenic), some plants have evolved the capacity to hyperaccumulate Tl, and for these species (or populations) a critical threshold level of 100 µg Tl g^−1^ dry weight (DW) in leaves has been set (van der Ent *et al.*, 2013). Thallium hyperaccumulators so far identified include few species: *Galium* sp., *Silene latifolia* (Caryophyllaceae), *Iberis intermedia* and *Biscutella laevigata* (Brassicaceae) (Escarré *et al.*, 2011; Corzo Remigio *et al.*, 2022*a*). *Biscutella laevigata* is the species with the highest recorded Tl concentrations in nature, capable of attaining 32 700 μg Tl g^−1^ DW in its leaves in the decommissioned Raibl mine in Italy (Fellet *et al.*, 2012) and ≤1490 μg Tl g^−1^ DW at Saint-Laurent-le-Minier in France (the accession used in this study, and here called Les Malines) (Escarré *et al.*, 2011). *Biscutella laevigata* L. has a widespread distribution across Europe and occurs on limestone rocky outcrops, but also occurs on (abandoned) Zn–Pb–Cd mine sites (Babst-Kostecka *et al.*, 2014). Thallium hyperaccumulation in *B. laevigata* depends on the genetic properties of the population and on local environmental conditions. It has been demonstrated that this species has a marked genetic plasticity that results in the fast differentiation of metallicolous populations able to tolerate and accumulate several metals, such as Zn, Pb and Tl (Fellet *et al.*, 2012; Pošćić *et al.*, 2013). The ecotype(s) established in calamine soils (i.e. soils enriched in Zn, Pb, Cd and Tl) have evolved a trait for Tl hyperaccumulation, e.g. *B. laevigata* subsp. *laevigata* from the former Zn–Pb mines in Cave del Predil (Italy) and the Zn–Pb mines at Saint-Laurent-le-Minier (France), whereas the non-metallicolous ecotype from the Tatra Mountains (Poland) does not hyperaccumulate Tl (Fellet *et al.*, 2012; Pošćić *et al.*, 2013).

A previous investigation on *B. laevigata* (Saint-Laurent-le-Minier accession) has shown that Tl concentration is highest in the intermediate leaves of the hyperaccumulating ecotype (16 100 μg g^−1^ DW) and an order of magnitude lower in the stem and roots (Corzo Remigio *et al.*, 2022*b*). The higher Tl concentrations in the intermediate and young leaves compared with old leaves suggest efficient phloem recycling. *Biscutella laevigata* from the Polish ecotype grown in a glasshouse experiment with calamine soil was reported to accumulate only a maximum of 588 μg Tl g^−1^ in its leaves in soils with 15.2–66.7 μg Tl g^−1^ DW and mainly stored excess metals in the old leaves (Wierzbicka *et al.*, 2017). At the organ level, Tl was shown to be localized in the blade and margins of the leaves (Corzo Remigio *et al.*, 2022*b*).

Plants of the Brassicaceae family are proposed to take up Tl via potassium (K^+^) channels and transporters, because K^+^ systems cannot discriminate between K^+^ and Tl^+^ owing to their monovalent similar ionic radii, resulting in indiscriminate uptake of Tl^+^ (Rader *et al.*, 2019). In a Tl:K competition experiment, it was shown that high-K (6 mm) treatment decreased shoot Tl concentrations by 50 %, but this happened only in some specific populations (Pošćić *et al.*, 2013). The same effect was not reproduced by Corzo Remigio *et al.* (2022a), who showed that at 10-fold lower K concentrations, K and Tl uptake were not significantly correlated, and that K neither inhibited nor stimulated Tl uptake by *B. laevigata*. The inconsistent interactions between Tl and K suggests that in *B. laevigata* this element might be taken up by modified K channels with higher affinity for Tl (Pošćić *et al.*, 2013), but these transporters remain to be identified in this species.

Several studies show that Tl speciation in plants is dominated by the monovalent state, as reported in *Brassica oleracea* var. *acephala*, where Tl(I) is present in aqueous and solid form, with minor contributions of Tl(III) (Corzo Remigio *et al.*, 2024). In *Sinapsis alba*, Tl(III) has been reported in minor quantities as an unstable compound in fresh samples (Sadowska *et al.*, 2016). The oxidation of Tl(I) to Tl(III) in plants is possible; although the latter is more toxic, the Tl trivalent forms thermodynamically stable glutathione and cysteine complexes (Krasnodębska-Ostręga *et al.*, 2012; Khan *et al.*, 2018). This trivalent organic complexation is a process that can explain Tl fractionation in plants, with higher levels of ε^205^Tl in the stem and lower levels in the soil and younger leaves, flowers and seeds (Rader *et al.*, 2019).

The extreme tolerance and bioaccumulation in *B. laevigata* trigger questions about the physiological processes related to uptake, distribution and sequestration of Tl in this species. The present study aimed to assess the differences in Tl tolerance and distribution between hyperaccumulating (Les Malines, France) and non-accumulating (Feltre, Italy) accessions corresponding to two ecotypes of the same species, and to resolve the tissue- and cellular-level distribution of Tl in *B. laevigata* using synchrotron X-ray fluorescence microscopy. These insights will accelerate the research endeavours into unravelling the genetic basis of exceptional Tl homeostasis in this species. In the future, this information will provide essential knowledge on what traits to focus on in domestication of this species for Tl-extraction purposes, given that phytoextraction is potentially an excellent method to remediate Tl-polluted soils while obtaining Tl products.

## MATERIALS AND METHODS

### Plant culture conditions

*Biscutella laevigata* seeds of two accessions were collected in the wild: the Les Malines accession originates from southern France (Saint-Laurent-le-Minier area, coordinates 43.935889 N, 3.671420 E) and the Feltre accession from northern Italy (coordinates 46.059815 N, 11.8290703 E). Seeds were germinated on perlite moistened with tap water, at a temperature of 23 °C and with a photoperiod of 16 h–8 h light–dark. Two-week-old seedlings were transferred into Nutriculture20 aeroponic systems (Nutriculture LTD, Skelmersdale, UK) filled with half-strength modified Hoagland’s formulation prepared with: K (3 mm as KNO_3_), Ca [2 mm as Ca(NO_3_)_2_·4H_2_O], P (1 mm as NH_4_H_2_PO_4_), N [8 mm as KNO_3_, Ca(NO_3_)_2_·4H_2_O and NH_4_H_2_PO_4_], Mg (0.5 mm as MgSO_4_·7H_2_O), Fe [40 µm as Fe(K)-HBED], Cl (1 µm as KCl), B (25 µm as H_3_BO_3_), Mn (2 µm as MnSO_4_·4H_2_O), Zn (2 µm as ZnSO_4_·7H_2_O), Cu (0.1 µm CuSO_4_·5 H_2_O), Mo (0.1 µm as Na_2_MoO_4_·2 H_2_O) and 2 mm MES [2-(*N*-morpholino)ethanesulfonic acid] buffer adjusted to pH 5.5 with KOH. The plants were cultivated with normal nutrient solution for 1 week. From week 2 to week 4, Tl was supplied in the form of Tl(I)carbonate (dissolved in 1 m HNO_3_) at three concentrations, 0 (control), 5 and 30 µm. The nutrient solution was kept at a pH of 5.8 ± 0.1, and a 20 % replacement was performed weekly. Plants were grown for 6 weeks in the same temperature and light conditions specified above. Additionally, seedlings for synchrotron analysis were cultivated on perlite soaked with the half-strength Hoagland’s solution containing 30 µm Tl and left growing for 1 week in the conditions mentioned above.

### Chemical analysis of plant material

Harvested plants were separated into young leaves, old leaves and roots, and the different organs were dried in a drying oven at 60 °C for 48 h. Plant organs were weighed to calculate the overall plant DW biomass. The organs were ground separately to a fine powder (<200 µm) in an analytical mill (IKA, Staufen, Germany). For microwave digestion, samples weighing 200 ± 5 mg were placed in teflon digestion tubes with 6 mL of 69 % HNO_3_ and 0.5 mL 35 % H_2_O_2_. The cycle was 2 min at 250 W, 2 min at 400 W, 1 min at 0 W, 2 min at 600 W and 33 min cooling using a StartD digestion system (Milestone, Sorisole, BG, Italy). After digestion, the samples were brought up to a volume of 20 mL with MilliQ water and filtered through Whatman 42 (Maidstone, UK) ashless filter paper. The measurement of trace elements was performed with a Spectro Arcos 2 ICP-AES (Ametek, Kleve, Germany).

### Analysis of genome size

The genome sizes (in picograms per 2*C*) of the *B. laevigata* accessions studied here were determined using flow cytometry performed by Plant Cytometry Services (Didam, The Netherlands).

### Synchrotron-based micro-X-ray fluorescence experimentation

The synchrotron-based micro-X-ray fluorescence (µXRF) experiments were undertaken at PETRA III (Deutsches Elektronen-Synchrotron DESY), a 6 GeV synchrotron radiation source, specifically at the hard X-ray microprobe experiment at the undulator beamline P06. P06 is equipped with a cryogenically cooled double-crystal monochromator with Si(111) crystals. Using different focusing optics, the X-ray beam can be focused down to sub-micrometre level. An ion chamber upstream of the sample is used to monitor the incoming flux, while a 500-µm-thick Si PIPS diode with 19-mm-diameter active area [PD300-500CB, Mirion Technologies (Canberra) GmbH, Germany] located downstream of the sample can be used to record the transmitted X-ray intensity to extract absorption data. Multiple XRF detectors allow for the measurement of X-ray fluorescence data. The incident X-ray energy was 16 keV for the whole experiment. A stack of 50 beryllium compound refractive lenses (CRLs) (RXOPTICS, Germany) and additional prefocusing was used to focus the X-ray beam down to 2.1 µm h × 3.6 µm (height × width), resulting in a flux of ~1.25^11^ photons s^−1^ in the focus. For XRF detection, both a Vortex ME4 in 45° geometry and a prototype 16-element SDD Ardesia detector (800-µm-thick chip with ~324 mm^2^ combined active area for all 16 elements; Politecnico Milano, Italy) (Utica *et al.*, 2021) in 315° geometry with Xspress 3 pulse processors were used.

### Data processing and statistical analyses

The XRF data were processed using non-linear least-squares fitting as implemented in PyMCA (Solé *et al.*, 2007), and figures were prepared in ImageJ (Schneider *et al.*, 2012) by changing the Lookup Table to ‘Fire’, adjusting of the maximum values and adding length scales. All variables were tested for homogeneity using Levene’s test for homogeneity of variance from the package car (v.4.3.2) (Fox and Weisberg, 2019) and for normality using the Shapiro–Wilk normality test from the package stats. Given that tested data turned out to be parametric, one-way ANOVA (*P* ≤ 0.05) followed by Tukey’s *post hoc* HSD test (two-tailed) was performed to detect significant differences among the three Tl treatments in the Les Malines accession, and Student’s *t*-test was used to detect a significant difference between control and 5 μm Tl in the Feltre accession. Statistical analyses were carried out using the software R v.4.3.2.

## RESULTS

### Plant growth performance in thallium dosing regimes

**Fig. 1.**
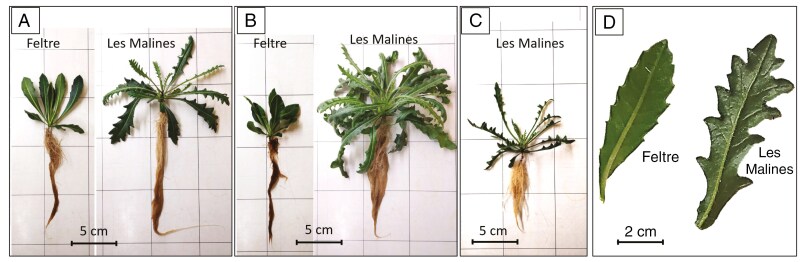
*Biscutella laevigata* accessions under different treatments. (A) 0 µm Tl. (B) 5 µm Tl. (C) 30 µm Tl (Feltre accession did not tolerate this treatment). (D) Differences in morphology of the leaves of the two accessions. The Les Malines (Tl-hyperaccumulating) accession has lobate leaves, whereas the Feltre (non-accumulating) accession has dentate leaves.

*Biscutella laevigata* plants were grown in aeroponics dosed with 0, 5 and 30 µm Tl (Fig. 1A–C). Plants from the two accessions have different leaf shapes and trichome distributions. The Les Malines accession is characterized by deeply lobated broad leaves with hard and scattered trichomes, whereas the Feltre accession has lanceolate smaller leaves with dense and soft smaller trichomes (Fig. 1D). The Feltre accession is distinctly less Tl tolerant than the Les Malines accession. In the control treatment, root biomass was similar in both Les Malines and Feltre accessions (0.27 ± 0.06 and 0.3 ± 0.1 g DW respectively), as was the shoot biomass (2.5 ± 0.8 and 2.7 ± 0.6 g DW respectively; Table 1). The shoot biomass of the Feltre accession markedly dropped in the 5 µm Tl treatment, to 1.1 ± 0.22 g DW compared with 6 ± 1 g DW of Les Malines. None of the plants from the Feltre accession survived in the 30 µm Tl treatment (Fig. 1C), and the shoot biomass dropped to 1.0 ± 0.1 g DW per plant in the Les Malines accession (Table 1). Rosette leaf area was also evaluated to assess plant health status. In general, the Les Malines accession had a greater leaf area compared with the Feltre accession (5-fold lower) in all treatments (Table 1).

**Table 1.** *Biscutella laevigata* growing parameters. Letters indicate significant differences for a given parameter between treatments (capital letters: Feltre accession; small letters; Les Malines accession). Student’s *T*-test was performed to detect significant differences in Feltre accession between control and 5 μM Tl treatment (*P* < 0.05). ANOVA, followed by Tukey HSD post-hoc test was performed detect significant differences among the three Tl treatments in Les Malines accession (*P* < 0.05). Values are means of three replicates ± standard deviation

Treatment (M Tl)	Accession	Root (g DW/plant)	Shoot (g DW/plant)	Leaf area (cm^2^/plant)
0 (Control)	Feltre	0.3 ± 0.1^A^	2.5 ± 0.8^A^	35 ± 9^A^
	Les Malines	0.27 ± 0.06^a^	2.7 ± 0.9^a^	210 ± 10^a^
5	Feltre	0.11 ± 0.03^B^	1.1 ± 0.2^B^	103 ± 6^B^
	Les Malines	1 ± 0.2^b^	6 ± 1^b^	490 ± 90^b^
30	Feltre	^_^	^_^	^_^
	Les Malines	0.18 ± 0.05^c^	1 ± 0.1^c^	110 ± 10^c^

### *Accumulation of thallium in* B. laevigata *accessions*

The *B. laevigata* accessions differed greatly in their Tl accumulation (Table 2). In the control conditions (0 µm Tl), plants from both accessions displayed a negligible amount of Tl (mean values of 3–5 μg Tl g^−1^), and at the 5 µm Tl dose level plants from the Feltre accession accumulated 107 ± 3 μg Tl g^−1^ in roots and 125 ± 6 μg Tl g^−1^ in old leaves. In comparison, plants from the Les Malines accession accumulated 37 ± 3 μg Tl g^−1^ in roots and 1750 ± 30 μg Tl g^−1^ in old leaves, demonstrating its stronger hyperaccumulating capacity. As further confirmation, the Tl shoot/root ratio (bioaccumulation factor) was 0.28 and 47 for Feltre and Les Malines accessions in the 5 µm Tl treatment. At the 30 µm Tl dose, the accumulation was ≤12 300 ± 200 and 4400 ± 200 μg Tl g^−1^ DW in old and young leaves, respectively. The concentrations of other micro-elements (Cu, Fe and Mn) were unremarkable and did not differ significantly between the plants from the two accessions, nor the treatments. The only exception to this trend was the concentration of Zn, which was 2-fold higher in the Les Malines accession in roots and old leaves irrespective of the treatment.

**Table 2.** *Biscutella laevigata* thallium and microelement concentration in plant organs. Stars indicate significant differences for a given element in a given organ between the two accessions after Student’s *T*-test (*P* < 0.05). Values are the mean of 3 replicates ± standard deviation

Treatment (µM Tl)	Accession	Plant organ	Cu (μg g^−1^DW)	Fe (μg g^−1^DW)	Mn (μg g^−1^DW)	Zn (μg g^−1^DW)	Tl (μg g^−1^DW)
0 (control)	Feltre	Roots	74 ± 2	990 ± 30	130 ± 10*	63 ± 2*	3.1 ± 0.4
		Old leaves	1.3 ± 0.1	50 ± 7	35 ± 2	75 ± 9*	3 ± 1
		Young leaves	5.1 ± 0.7	61 ± 6	18.0 ± 0.3	49 ± 5	4.3 ± 0.8
	Les Malines	Roots	70 ± 2	950 ± 30	80 ± 8	236 ± 4	3 ± 1
		Old leaves	1.1 ± 0.2	44 ± 6	48 ± 3	120 ± 10	3.8 ± 0.4
		Young leaves	1.5 ± 0.2	45 ± 5	18.0 ± 0.4	59 ± 8	5.5 ± 0.7
5	Feltre	Roots	65 ± 3	2180 ± 190	92 ± 9	98 ± 9*	107 ± 3*
		Old leaves	1.8 ± 0.1	54 ± 7	37 ± 2	110 ± 10*	125 ± 6*
		Young leaves	3.1 ± 0.4	53 ± 5*	29.0 ± 0.7*	62 ± 6	31 ± 2*
	Les Malines	Roots	72 ± 2	2710 ± 80	120 ± 10	235 ± 7	37 ± 3
		Old leaves	2.3 ± 0.1	60 ± 10	47 ± 3	160 ± 20	1750 ± 30
		Young leaves	4.4 ± 0.7	75 ± 8	20.0 ± 0.4	51 ± 6	1750 ± 20
30	Les Malines	Roots	55 ± 2	1640 ± 70	66 ± 7	343 ± 7	440 ± 30
		Old leaves	2.5 ± 0.3	80 ± 10	49 ± 3	110 ± 10	12,300 ± 200
		Young leaves	5.5 ± 0.8	90 ± 10	27 ± 2	61 ± 6	4400 ± 200

### Root thallium distribution

**Fig. 2.**
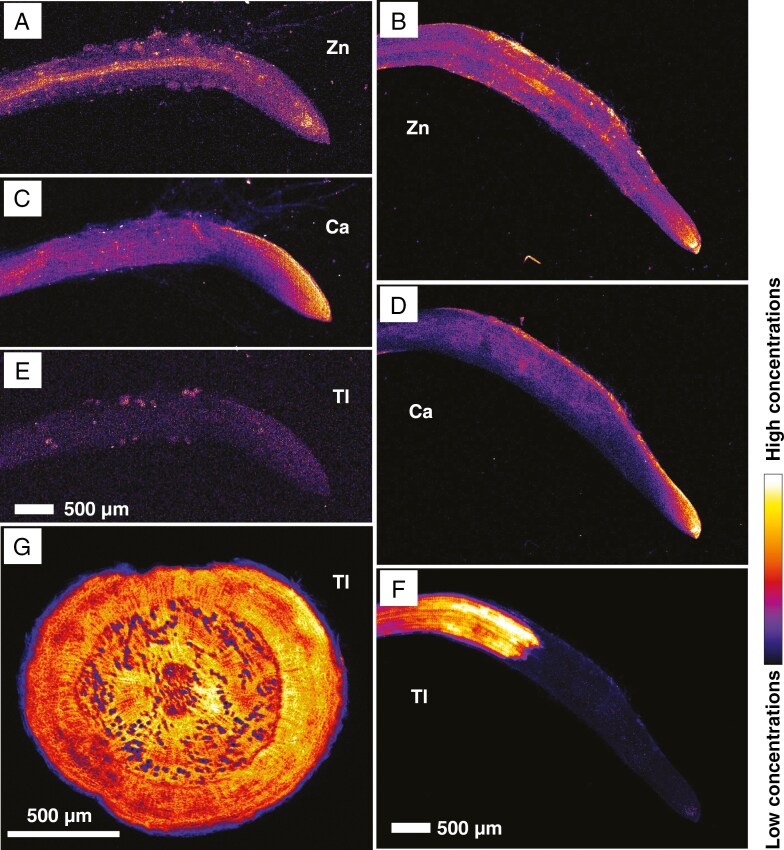
Synchrotron micro-X-ray fluorescence elemental maps showing the distribution of Zn (A, B), Ca (C, D) and Tl (E–G) in intact root tips and tap root (G) of *Biscutella laevigata* dosed at 5 µm Tl. (A, C, E) Feltre accession. (B, D, F, G) Les Malines accession. The scans measure 4.64 mm × 3.5 mm (A, C, E) or 2.72 mm × 1.23 mm (B, D, F), with a resolution of 7 or 3 µm, respectively, a dwell time of 5 ms and a total acquisition time of 31 or 27 min, respectively. (G) The scan measures 2.2 mm × 2 mm, resolution 8 µm, dwell time 5 ms and total acquisition time 2 h 8 min.

For adventitious young roots, Zn (Fig. 2A, B) and Ca (Fig. 2C, D) distribution did not differ between the two accessions. Zinc was distributed mainly in the central stele and for Les Malines accession in the differentiation zone at the tip, whereas the Ca was present mostly in the root tip, especially on the side having predominant cellular distension in both accessions. Thallium was strongly enriched in the central part of the root before the differentiation zone in the Les Malines accession, whereas such enrichment was not present in the Feltre accession (Fig. 2E, F). The main tap root (Fig. 2G) of the Les Malines accession showed abundance of Tl in every tissue, from the xylem to the phloem and cortical zone, with the only exception being the epidermis, which appeared to be very low in Tl, as in the small adventitious roots.

### Stem thallium distribution

**Fig. 3.**
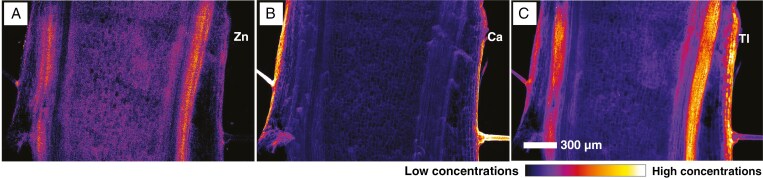
Stem longitudinal section of *Biscutella laevigata* Les Malines accession dosed at 5 µm Tl. (A) Zn distribution. (B) Ca distribution. (C) Tl distribution. Scan measures 2.0 mm × 1.5 mm, resolution 5 µm, dwell time 5 ms and total acquisition time 78 min.

In the longitudinal stem section (Fig. 3) of the Les Malines accession, Zn is mostly co-localized with Tl, except for the epidermal layer where its presence is negligible (Fig. 3A). Calcium is found on the outer part of the epidermis and is the main constituent of trichomes (Fig. 3B). Tl is concentrated mostly in the phloem cells and in the epidermal layer (Fig. 3C). A minor concentration can also be detected in the xylem, whereas the pith does not contain Tl.

### Thallium distribution in seeds and seedlings

**Fig. 4.**
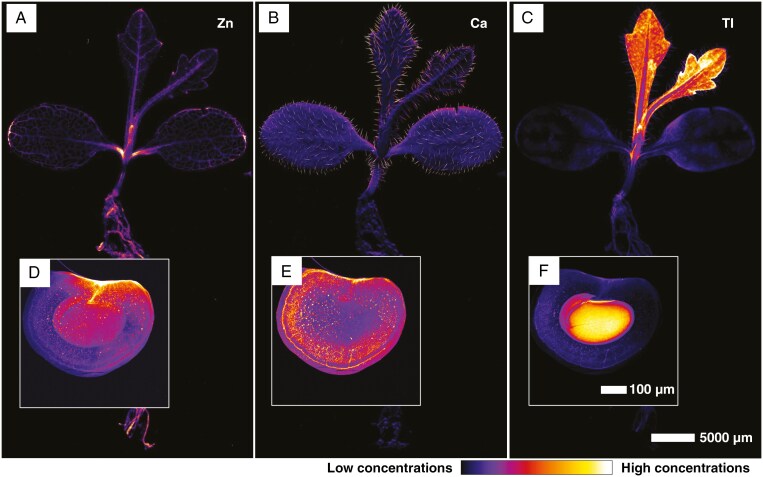
Seeds and seedlings of *Biscutella laevigata* (Les Malines accession), grown on perlite dosed with 30 µm Tl. (A, D) Distribution of Zn. (B, E) Distribution of Ca. (C, F) Distribution of Tl. (A–C) Scans measure 52.96 mm × 29.22 mm, with a resolution of 15 µm, a dwell time of 5 ms and a total acquisition time of 9 h 51 min. (D–F) Scans measure 13.44 mm × 6.12 mm, with a resolution of 8 µm, a dwell time of 5 ms and a total acquisition time of 1 h 53 min.

At the plant level (whole live seedling), trichomes are highlighted by the presence of Ca, whereas Zn is localized in the vascular bundles of leaves and cotyledons (Fig. 4A, B). The thallium concentration is highest in the youngest leaf and depleted in the cotyledons. Again, a clear enrichment in Tl in vascular strand, close to cotyledon area can be seen. Compared with the rest of the plant, the roots are depleted in Tl (Fig. 4C). In intact seed capsules, Zn is distributed in the vasculature of the capsule, where it is attached to the petiole (Fig. 4D), and Ca occurrs in the hilum and in hotspots on the surface of the capsule (Fig. 4E). Thallium is concentrated in the cotyledon and, to a lesser extent, the hypocotyl of the embryo (Fig. 4F). Thallium depletion in the cotyledons after germination (Fig. 4C) demonstrates active phloem mobilization of this element.

### Thallium distribution in leaves and petioles

**Fig. 5.**
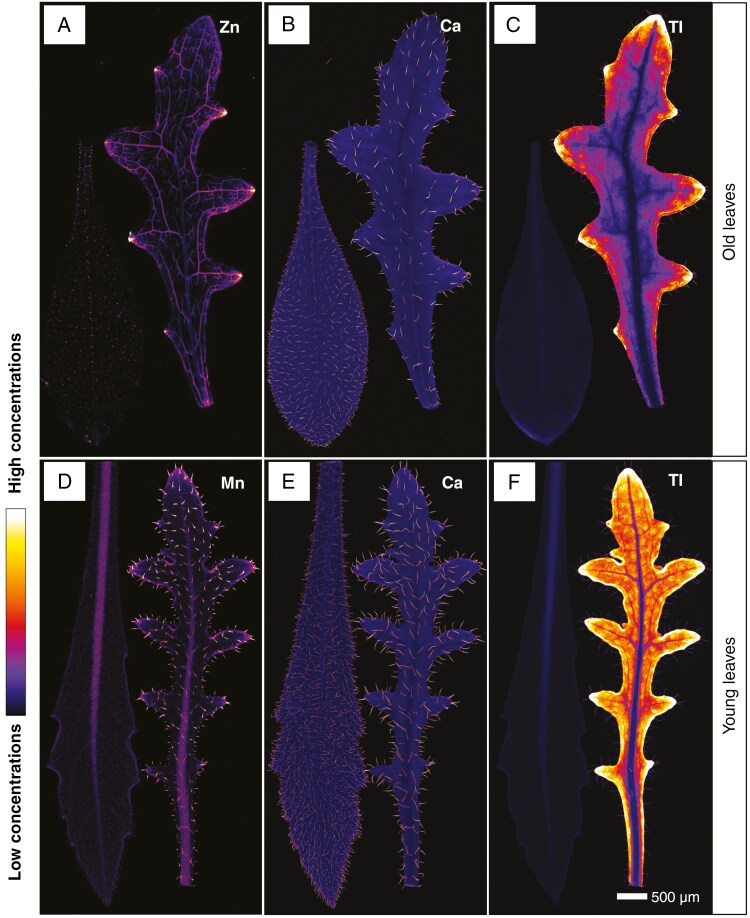
Distributions of Mn (D), Zn (A), Ca (B, E) and Tl (C, F) in old (top) and young (bottom) leaves of *Biscutella laevigata*. Feltre and Les Malines accessions are on the left and right, respectively, of each panel and dosed at 5 µm Tl. The scans measure 71.61 mm × 35 mm (top) and 75 mm × 38 mm (bottom), with a resolution 20 µm, a dwell time of 5 ms and a total acquisition time of 8 h 11 min and 10 h 12 min, respectively.

In the whole leaves (Fig. 5) of the Les Malines and Feltre accessions, Zn and Mn (Fig. 5A, D) are more concentrated in the former accession. Zinc can be found in the vasculature of the leaf, whereas Mn is found in the trichome bases and in the trichome itself. In the Les Malines accession, Mn is also enriched around the margins of the leaf tips. Both accessions have similar distributions of Ca, which is localized in the unicellular trichomes (Fig. 5B, E). The distribution of this element is similar in old and young leaves. There is no notable Tl enrichment in the leaves of the Feltre accession, but in the Les Malines accession Tl is strongly enriched in the margins of the leaf tips (Fig. 5C, F) and depleted in the main and secondary veins. Comparing young leaves with old leaves (5 µm treatment) in the Les Malines accession, Tl is more enriched towards the centre of the leaf blade in young ones.

**Fig. 6.**
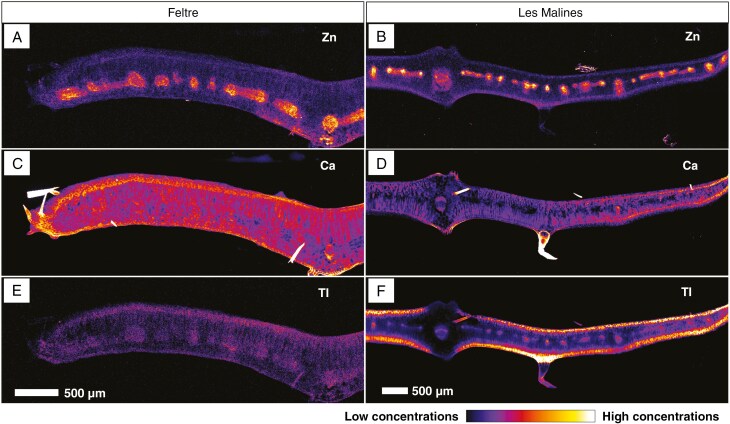
Distributions of Zn (A, B), Ca (C, D) and Tl (E, F) in *Biscutella laevigata* leaf cross-sections. Left and right panels are Feltre and Les Malines accessions, dosed at 5 µm Tl, respectively. Scans measure 4 mm × 1.7 mm, resolution 6 µm, dwell time 5 ms and total acquisition time 2 h 5 min.

In leaf cross-sections of the two *B. laevigata* accessions dosed with 5 µm Tl, zinc shows a very similar distribution in both accessions, with notable enrichment in the vascular bundles (secondary veins) and in the central (main) vein (Fig. 6A, B), as previously observed for the whole leaf. The distribution of Ca is comparable between the two accessions, with enrichment in the epidermis and mesophyll, and particularly in the trichomes. However, concentrations differ between accessions, with the Feltre accession being more enriched overall than the Les Malines accession (Fig. 6C, D). The thallium distribution in the two accessions is similar, but the Tl concentrations are about two orders of magnitude lower in the Feltre accession compared with the Les Malines accession. In line with the whole leaf scan, the cross-section shows Tl enrichment in both (abaxial and adaxial) epidermides, especially at the base of the trichome and to a less extent in the secondary veins (Fig. 6E, F).

**Fig. 7.**
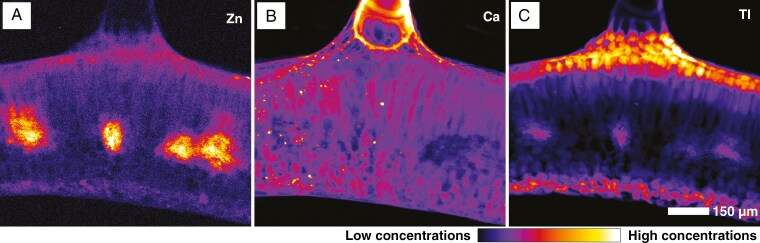
Synchrotron micro-X-ray fluorescence elemental maps showing the distribution of Zn (A), Ca (B) and Tl (C) in a leaf cross-section of *Biscutella laevigata* (Les Malines accession dosed at 30 µm Tl). The scan measures 3.25 mm × 2.28 mm, with a resolution 2 µm, a dwell time of 5 ms and a total acquisition time of 2 h 41 min.

**Fig. 8.**
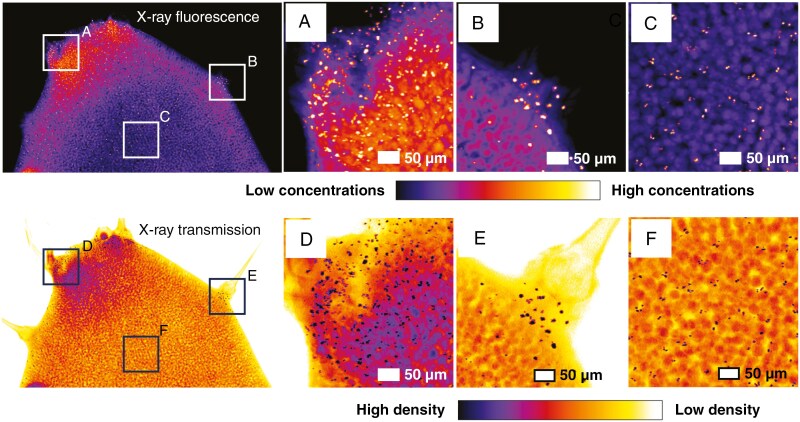
Micro-X-ray fluorescence (top) and transmission (bottom) distribution map of Tl hotspots in a leaf tip of *Biscutella laevigata* (Les Malines accession dosed at 30 µm Tl). The micro-X-ray fluorescence scan measures 0.89 mm × 0.82 mm, with a resolution of 2 µm, a dwell time of 5 ms and a total acquisition time of 18 min.

The high-resolution scan (Fig. 7) of a leaf cross-section dosed at 30 µm Tl shows that Zn has a very distinct distribution and is concentrated mostly in the vascular bundles, probably phloem and to a lesser extent in the photosynthetic parenchyma (Fig. 7A). Detailed observation of the trichome area clarifies that Ca is in the intercellular space of the trichome basal cells and in the cell wall of the trichome itself (Fig. 7B). Thallium is concentrated in the adaxial and axial sides of the leaf, in the vacuoles of epidermal and at much lower concentration in palisade photosynthetic tissues. The highest concentrations are found at the trichome base (Fig. 7C). A detailed scan of a leaf tip confirms Tl occurring in the vacuoles of the epidermal and parenchyma cells but reveals the presence of conspicuous minute Tl granules, spread most densely near the foliar margin and trichome base (Fig. 8A, B). X-ray transmission images reveal that these granules are of very high density and appear to have a polygonal/crystalline structure (Fig. 8D, E).

**Fig. 9.**
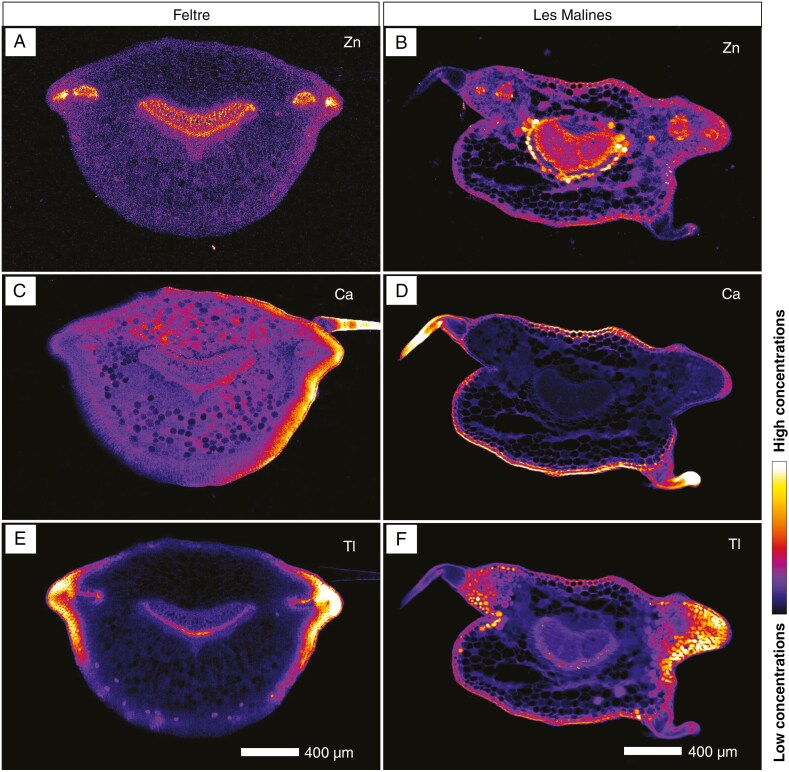
Synchrotron micro-X-ray fluorescence elemental maps showing the distribution of Zn (A, B), Ca (C, D) and Tl (E, F) in a petiole cross-section of *Biscutella laevigata*, dosed at 5 µm Tl. Left and right panels are Feltre and Les Malines accessions, respectively. Scans measure 2.0 mm × 1.5 mm, resolution 4 µm, dwell time 5 ms and total acquisition time 78 min.

In the petiole cross-sections of the *B. laevigata* (dosed at 5 µm Tl), Zn distribution is similar in both accessions, and predominant in the xylem of the central and lateral vasculatures. In the Les Malines accession, some Zn enrichment is found in apoplastic spaces between epidermal cells (Fig. 9A, B). In both accessions, Ca is concentrated in the trichomes and in the epidermal layer, and in the Les Malines accession, Ca is visibly enriched in the apoplastic space under the cuticle and around the epidermal cells as also observed for Zn (Fig. 9C, D). In the Les Malines accession, Tl is localized in the vacuoles of the adaxial and abaxial epidermal cells, and especially in the regions where the leaf blade will originate, which also host trichomes. A similar distribution can be found in the Feltre accession despite the general Tl concentration being lower and a minor enrichment in the epidermal cell being present (Fig. 9E, F).

## DISCUSSION

Thallium tolerance and accumulation is distinctly different between the Les Malines and Feltre accessions. How genetically close these two *B. laevigata* accessions are is presently unknown. A study on intraspecific genetic variation in *B. laevigata* grouped the Les Malines and Feltre accessions as both belonging to the tetraploid (2*n*2 = 36) *B. laevigata* subsp. *laevigata* taxonomic group (Tremetsberger *et al.*, 2002). However, flow cytometric data on the two accessions used showed the Feltre accession having a genome size twice that of the Les Malines accession (256 and 125 pg per 2*C*, respectively), suggesting that they are phylogenetically distinct and probably distant, in line with their geographical distance. This is supported by the obvious morphological differences between plants of these accessions, with the Les Malines accession having deeply lobate foliar margins and larger uniseriate trichomes, in comparison to the Feltre accession that has dentate margins and small, soft trichomes (Raffaelli and Baldoin, 1997). Nevertheless, this species is a model for autopolyploidization, which occurs frequently under environmental pressure (i.e. metallicolous soil conditions) (Babst-Kostecka *et al.*, 2016) and could mean that tetraploid accessions are closely related to diploid ancestors. The differences in Tl accumulation and tolerance between the two accessions are remarkable, with the Feltre accession tolerating a maximum of 5 μm Tl, whereas the Les Malines accession tolerates ≤10-fold higher levels (Corzo Remigio *et al.*, 2022*b*). Interestingly, although the 5 μm Tl treatment led to a strong growth reduction in the Feltre accession, with shoot biomass (66 % lower compared with control, from 2.5 to 1.1 g DW per plant), it has, on the contrary, a growth-promoting effect in Les Malines accession plants (+105 % compared with control, from 2.7 to 6 g DW per plant). This phenomenon has also been observed previously by Corzo Remigio *et al.* (2022*b*).

In terms of hyperaccumulation, the two accessions showed opposite phenotypes when grown with 5 μm Tl. The Feltre accession accumulated only 125 μg g^−1^ DW in old leaves, whereas the Les Malines accession accumulated >100-fold more in the same organ (1750 μg g^−1^ DW). The shoot concentration recorded in the Feltre accession was in line with that of a known non-accumulating *B. laevigata* Polish population (588 μg Tl g^−1^ DW) (Wierzbicka *et al.*, 2017), although these data were collected on plants grown in natural soils. The ICP-AES bulk analysis on aerial parts did not show differences in Tl content between young and old leaves in the Les Malines accession (1750 μg g^−1^ DW). However, synchrotron images demonstrated that in young leaves Tl concentrations are higher than in old leaves. The depletion of Tl from the centre towards the edge of old leaves suggests efficient phloem remobilization of this element towards actively growing organs. This is confirmed by the µXRF analysis of seeds and seedlings: Tl is mobilized from cotyledons and reallocated in the young leaves during seedling growth. Unlike other hyperaccumulators (i.e. selenium), in which newly formed leaves are the richest in the accumulated element (van der Ent *et al.*, 2023), *B. laevigata* has the highest Tl enrichment in young-to-intermediate leaves, whereas the newly formed ones have lower concentrations. In *Brassica juncea* also, higher Tl concentrations are in the leaves, and for new tissues the concentration is lower, e.g. seed pods, flowers; Tl translocation and redistribution in plant growth was confirmed with the fractionation of Tl isotopes, where ^205^Tl is higher in new tissues and lower in more mature tissues (Rader *et al.*, 2019). Thallium is widespread in the vascular tissues and the cortex, but not the epidermis, in both adventitious roots and the main root of the Les Malines accessions. In the Feltre accession, fine lateral roots are extremely Tl depleted, which might be the consequence of root barrier mechanisms aimed at preventing Tl uptake. The longitudinal stem section of the Les Malines accession showed phloem Tl enrichment, probably as a direct consequence of the translocation of this element from old leaves to young leaves in the rosette.

Overall, the synchrotron µXRF data show that Tl is localized in the vacuoles of epidermal and mesophyll cells, especially underneath trichomes and in trichome basal cells. In addition, Tl occurs in minute specks that appear to be solid crystalline deposits that could contain up to ~40 wt% Tl and are 3–5 µm in size. These Tl crystals with a similar size were also observed in *Brassica oleracea* var. *acephala*, wherein Tl(I) predominates in aqueous and solid form (Corzo Remigio *et al.*, 2024). The formation of insoluble Tl grains might be interpreted as a detoxification mechanism but could also be a passive process in which Tl salts are precipitated owing to the combination of high concentrations and local evapotranspiration; this mechanism was observed in halophytes (Ciccarelli *et al.*, 2010). The observation that these grains often appear in pairs leads to the hypothesis that their precipitation happens specifically in the guard cells of stomata. Another option is that Tl-rich crystals could be formed by guttation from hydathodes; in this case, salt deposits are left behind in the opening, and this also explains why crystal occurrence is predominant around the foliar margins, similarly to Mn and Ni hyperaccumulators (Mizuno *et al.*, 2002).

## Conclusion

The Feltre and Les Malines accessions of *B. laevigata* showed opposite phenotypes in terms of accumulation of Tl tolerance, with the former being sensitive to this element, whereas the latter accession is a strong hyperaccumulating one. The Feltre accession seems to limit Tl uptake in the roots; however, µXRF elemental maps have demonstrated that, once in the plant, Tl is handled in a similar way by both accessions (stored in the same tissues). Because of its fast growth and opposite expression of Tl accumulation traits, *B. laevigata* is a useful model species in which to study Tl hypertolerance and hyperaccumulation. Furthermore, as a member of the Brassicaceae family, which includes intensively studied Ni/Cd/Zn hyperaccumulators (*Noccaea caerulescens* and *Arabidopsis halleri*), it is a particularly attractive target for further investigations. In the future, it will be crucial to undertake a detailed assessment of the species *B. laevigata* subsp. *laevigata* complex to establish the genetic relatedness of accessions collected across southern Europe, based on regular chloroplast (e.g. matK) and nuclear (e.g. ITS) markers. After that, a selection of genetically close sensitive, tolerant and hyperaccumulating accessions can be chosen for transcriptomics investigations. Finally, the next nexus in our understanding will be levered from whole-genome sequencing to provide insights into the molecular mechanisms that distinguish the Tl-hyperaccumulating *B. laevigata* accession(s) from the non-accumulating ones.

## Supplementary Material

mcae115_suppl_Supplementary_Material

## Data Availability

The data that support this study will be shared upon reasonable request to the corresponding author.
