# Supplementary material for: Uncovering the physiology and distribution of thallium in Tl-hyperaccumulating and Tl-sensitive populations of Biscutella laevigata L
Source: Ann Bot. 2024 Sep 28;134(5):803–14. doi: 10.1093/aob/mcae115 (PMC11560374; doi:10.1093/aob/mcae115)
Supplement: mcae115_suppl_Supplementary_Material [file mcae115_suppl_supplementary_material.docx]

Laboratory of Genetics

Radix West, Building 107

Droevendaalsesteeg 1

6708 PB Wageningen

The Netherlands

11 April 2024

To the Editor of Annals of Botany

Dear Editor,

We are pleased to submit our manuscript “Uncovering the physiology and distribution of thallium in Tl-hyperaccumulating and Tl-sensitive populations of Biscutella laevigata L.” for consideration by Annals of Botany.

Thallium is extremely toxic to all lifeforms and emerging as a pollutant in geochemical anomalies and from industrial point sources. Plants in the Brassicaceae family, including edible crops, have enhanced capacity for thallium accumulation, even from soils with relatively low thallium status. The most extreme thallium hyperaccumulator known to date is *Biscutella laevigata* capable of attaining >32,000 mg/kg thallium in its leaves. Intriguingly, this trait occurs only two populations of this otherwise widespread taxon. The extreme tolerance and bioaccumulation in certain populations of Biscutella laevigata trigger questions about the physiological processes related to uptake, distribution, and sequestration of thallium in this species, which this studied aim to elucidate by comparing a hyperaccumulating and non-accumulating accession pair.

We used synchrotron X-ray imaging to elucidate the distribution of thallium in *B. laevigata* at cellular level. The insights generated in this study will accelerate the research endeavour into unravelling the genetic basis of exceptional thallium regulation in this species. It will also help future breeding efforts to improve the thallium accumulation in *B. laevigata* for phytoextraction purposes.

Our main findings pointed out that the hyperaccumulating and non-accumulating accessions showed opposite phenotypes in terms of thallium tolerance accumulation with the latter limiting thallium uptake in the roots. The genome size of the two used population is also different, in line with their geographical isolation. The MicroXRF elemental maps have demonstrated that, once in the plant, thallium is handled in a similar way by both accessions (stored in the same tissues). The synchrotron µXRF analysis revealed that thallium is localised in the vacuoles of epidermal cells, and thallium occurs in solid crystalline deposits (3–5 µm in size) of exceptional thallium enrichment (~40 wt% thallium) that are mainly found in foliar margins and under trichome bases. This type of internal detoxification mechanism has never been shown before and we discuss its implications.

Because of its fast growth and opposite expression of its thallium accumulation trait, Biscutella laevigata makes for a useful model species to study thallium hypertolerance and hyperaccumulation.

Thank you for your consideration of this manuscript, and we look forward to your response.

Yours sincerely,

Dr Antony van der Ent

Laboratory of Genetics, Wageningen University & Research. E-mail: antony.vanderent@wur.nl
